# 602 Foot burns in diabetic patients: A single center experience

**DOI:** 10.1093/jbcr/irac012.230

**Published:** 2022-03-23

**Authors:** Katherine J Choi, Justin Gillenwater, Christopher H Pham, Clifford C Sheckter, Zachary J Collier, Justin Dang, Samantha Huang, Haig A Yenikomshian, Warren L Garner

**Affiliations:** University of California Los Angeles, Los Angeles, California; USC/LAC+USC Medical Center, Los Angeles, California; University of Southern California, Cypress, California; Stanford University, Stanford, California; Keck School of Medicine, University of Southern California, Los Angeles, California; Sidney Kimmel Medical College at Thomas Jefferson University, Costa Mesa, California; USC, Los Angeles, California; USC/LAC+USC Medical Center, Los Angeles, California; USC, Los Angeles, California

## Abstract

**Introduction:**

The most significant sequelae of foot burns in diabetic patients is a non-healing wound that results in a diabetic foot ulcer, which has been a predictor for need for amputation and mortality. Even minor amputations in patients with diabetes have a significant mortality rate. Our systemic review found that when 60% of a diabetic patient cohort with foot burns was managed by skin grafting, 29% subsequently required amputations, which is concerning. The practice at our regional burn center has been to manage patients with foot burns and diabetes non-operatively with daily dressing changes, and we believe that this may result in better functional outcomes, ambulatory status, and lower amputation rates.

**Methods:**

A retrospective review of patients with diabetes and foot burns admitted to an ABA verified regional burn center was conducted. The primary outcome was amputation. Secondary outcomes were ambulatory status, wound closure, and infection. Rank sum and fisher exact tests were performed to compare patient demographics, comorbidities, uncontrolled DM (A1c >9%), and burn characteristics between patients who were treated surgically and those who received daily wound care only. These associations were subsequently evaluated with odds ratios (OR) and 95% confidence intervals (CI). A multivariable logistic regression was performed to evaluate for possible differentiating factors that resulted in maintaining ambulatory status at completion of burn care. Statistical significance was defined as p< .05.

**Results:**

Of 75 patients identified, median TBSA burned was 2% (IQR 2), and 61% (n=46) had full thickness burns. Mean A1c at admission was 9% (SD 2). In terms of management, 9% (n=7) were treated with debridement and/or skin grafting, and 9% (n=7) later required lower extremity amputations. Infection during first admission developed in 8% (n=6). At completion of burn care, 73% had normal or same ambulatory status, and older patients were less likely to maintain ambulatory status (p=.009, OR=0.92, 95% CI=0.86-0.98). Median time to wound closure was 95 (IQR 130) days, and 12% (n=9) of wounds never fully closed. Burn depth (second vs third degree), burn location (plantar burn vs other areas), uncontrolled DM, and surgical treatment did not result in a statistically significant difference in maintaining ambulatory status at completion of burn care.

**Conclusions:**

Diabetic patients with foot burns are best managed non-operatively with daily dressing changes, and should be allowed to heal secondarily. This may result in a longer time to close the wounds, but the amputation rates were much lower when compared to surgical management. However, 5% of our cohort developed diabetic foot ulcers at completion of burn care, which is a complex disease process that carries a dire prognosis.

